# Successful conservative management of a delayed duodenal perforation following displacement and removal of a pancreatic duct stent

**DOI:** 10.1055/a-2977-8023

**Published:** 2026-10-28

**Authors:** Shaofei Wang, Weiping Zhang, Liping Zhang, Qiming Huang, Ying Chen, Qiao Mei, Junjun Bao

**Affiliations:** 1 Department of GastroenterologyXuancheng City Central HospitalXuanchengAnhuiChina; 2 Department of Gastroenterology 36639 The First Affiliated Hospital of Anhui Medical UniversityHefeiAnhuiChina; 3 Department of GastroenterologyFirst Affiliated Hospital of Anhui Medical UniversityHefeiAnhuiChina; 4 Department of Endocrinology 117958 Suzhou Municipal HospitalSuzhouChina; 5 Gastroenterology 36639 First Affiliated Hospital of Anhui Medical UniversityHefeiAnhuiChina

**Keywords:** pancreatobiliary (ERCP/PTCD), ERC topics, endoscopic ultrasonography, pancreas

A 57-year-old man with common bile duct stones and uremia on long-term hemodialysis underwent therapeutic endoscopic retrograde cholangiopancreatography (ERCP). Because of repeated unintended pancreatic duct cannulation, a prophylactic pancreatic duct stent was placed after stone extraction. During deployment, the stent migrated deeply into the pancreatic duct due to excessive duodenal peristalsis ( Fig. 1 ). Initial retrieval using a balloon catheter and foreign-body forceps failed.

**Fig. 1:**
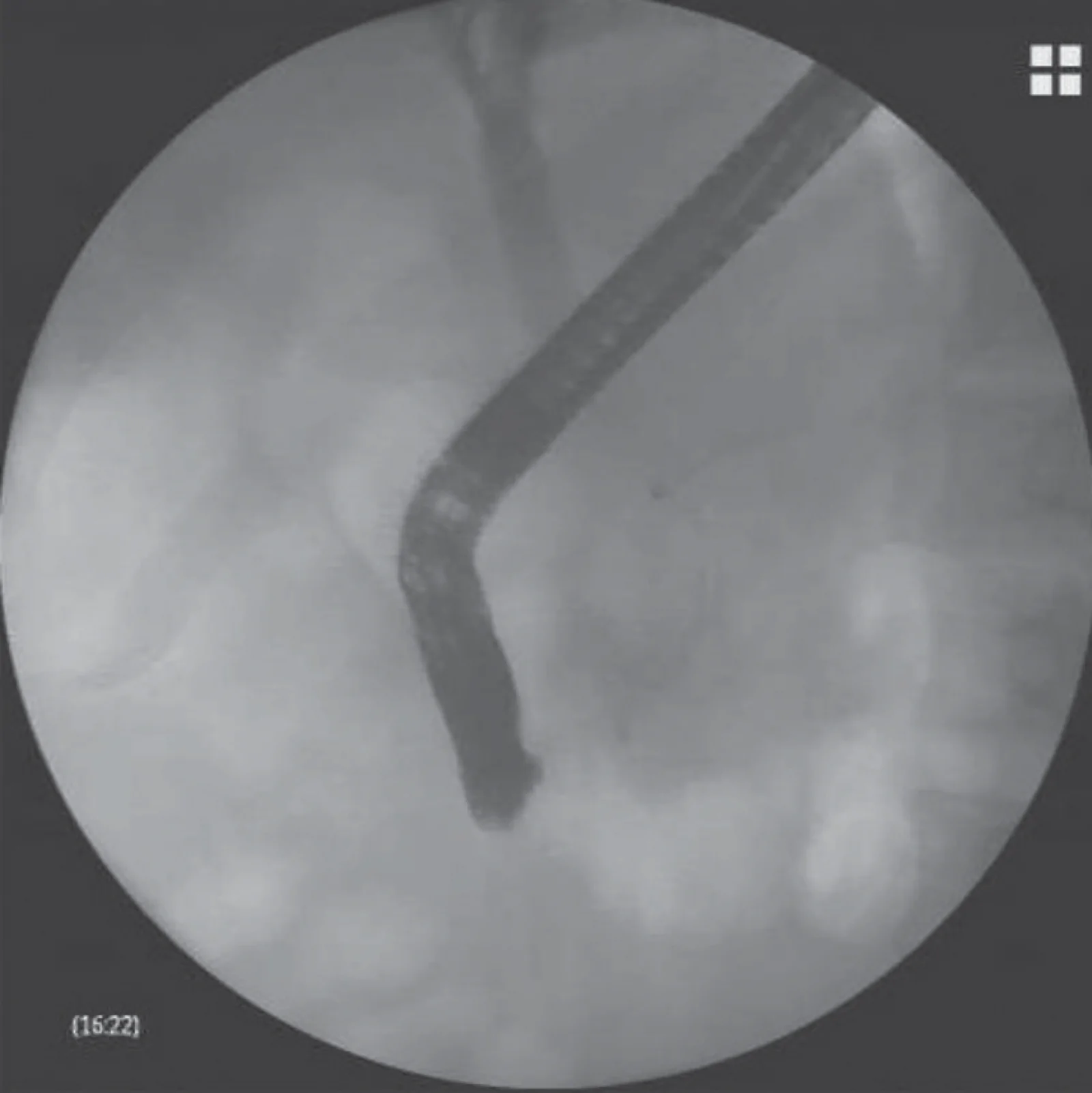
An X-ray image showing the intrapancreatic migration of the pancreatic stent.

Fifteen days later, repeat ERCP under general anesthesia successfully retrieved the migrated stent with a snare, followed by the placement of a new 5Fr-9cm single-pigtail pancreatic duct stent ( Fig. 2 ). Mild postoperative upper abdominal discomfort resolved with conservative treatment.

**Fig. 2:**
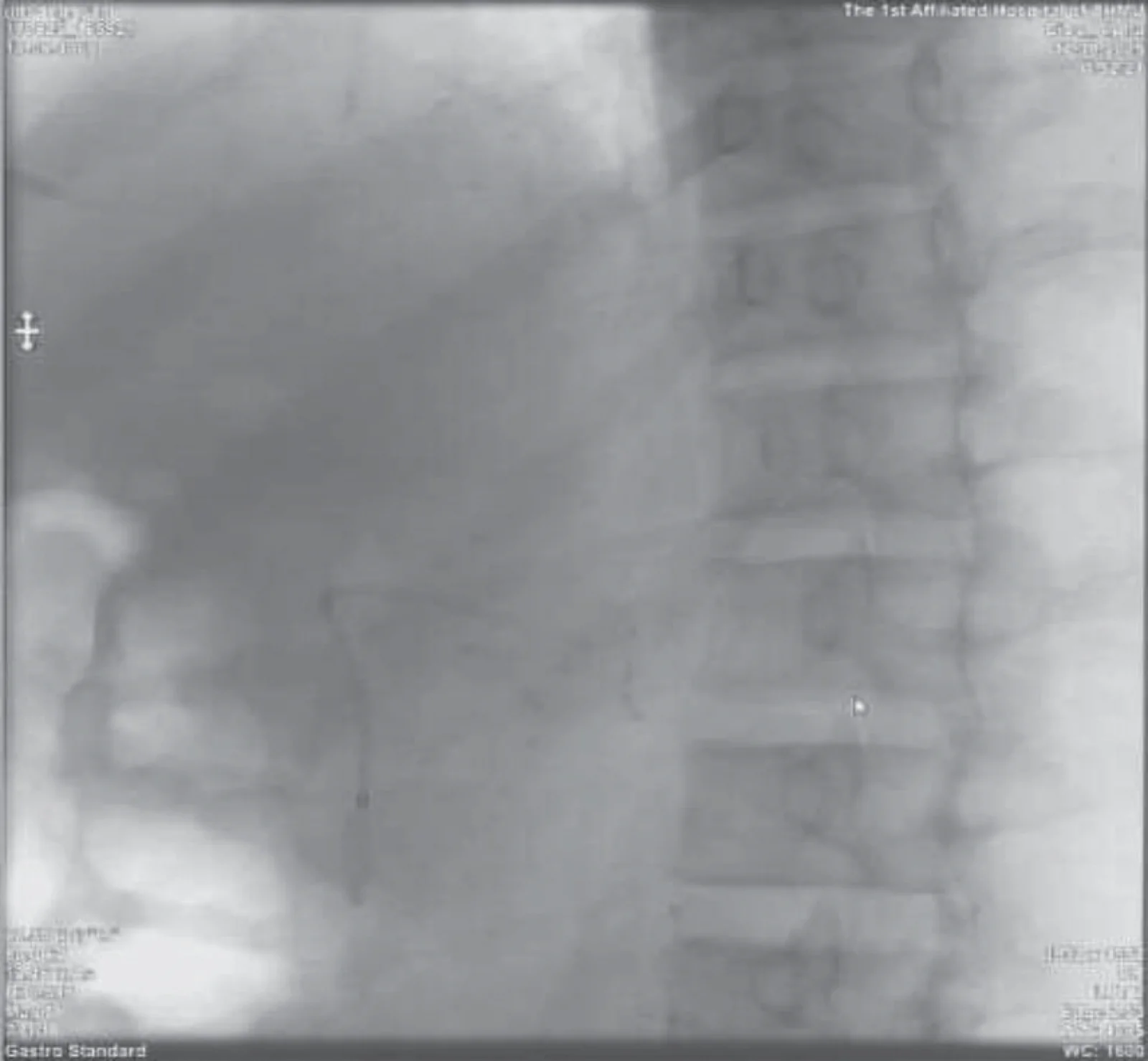
An X-ray image showing a newly placed 5Fr-9cm single-pigtail pancreatic duct stent.

Approximately 2 weeks after stent removal, the patient developed sudden severe abdominal pain. Computed tomography demonstrated peripancreatic fluid collections with gas, suggesting gastrointestinal perforation ( Fig. 3 ). Emergency percutaneous drainage and ERCP were performed. Although no contrast leakage was identified, the purulent material was aspirated from a periampullary diverticulum. Given the high suspicion of Stapfer type II duodenal perforation, a covered biliary metal stent was placed, and a nasojejunal tube was inserted for enteral nutrition ( Fig. 4 ).

**Fig. 3:**
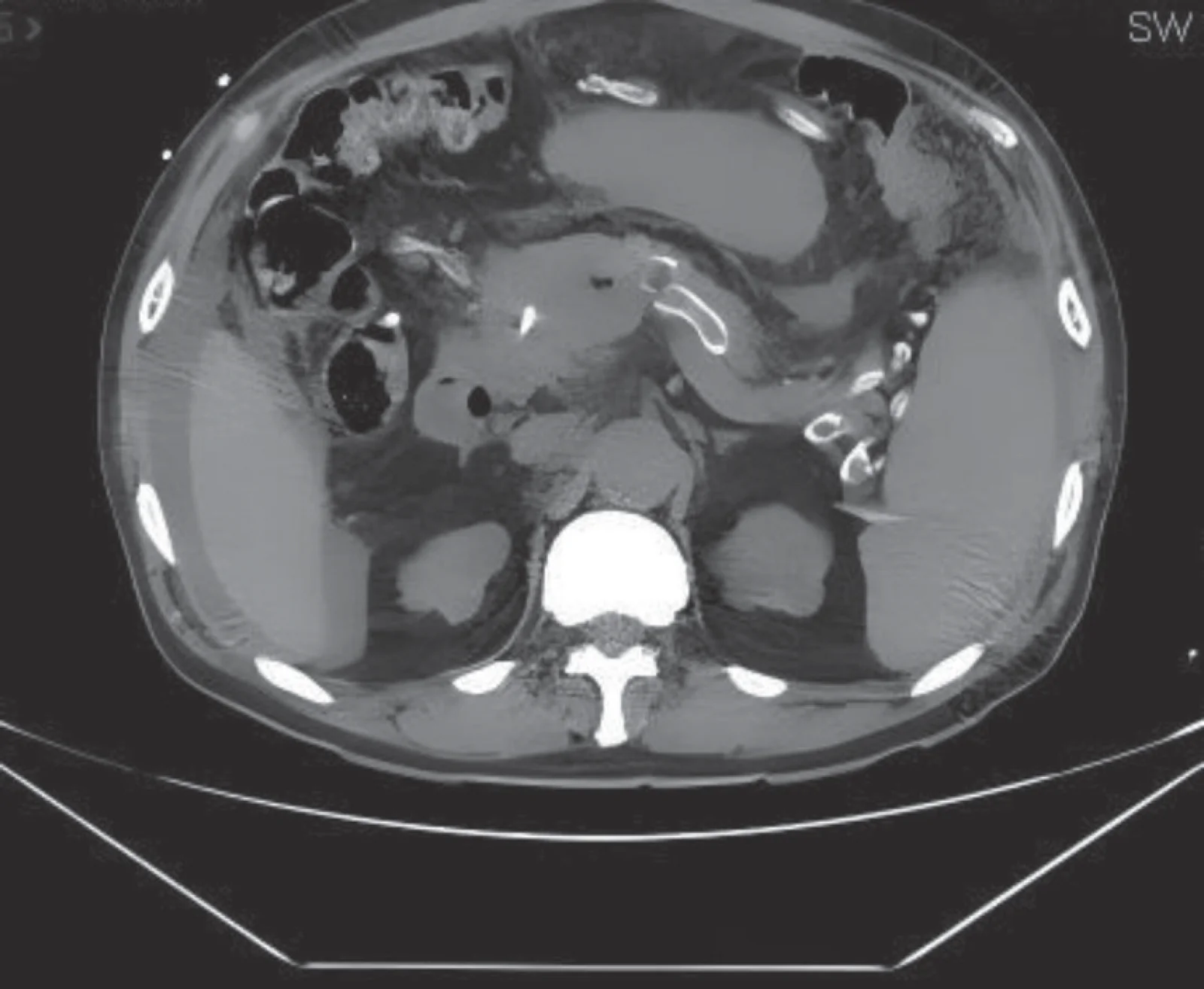
An emergency computed tomography image demonstrating gastrointestinal perforation with peritonitis.

**Fig. 4:**
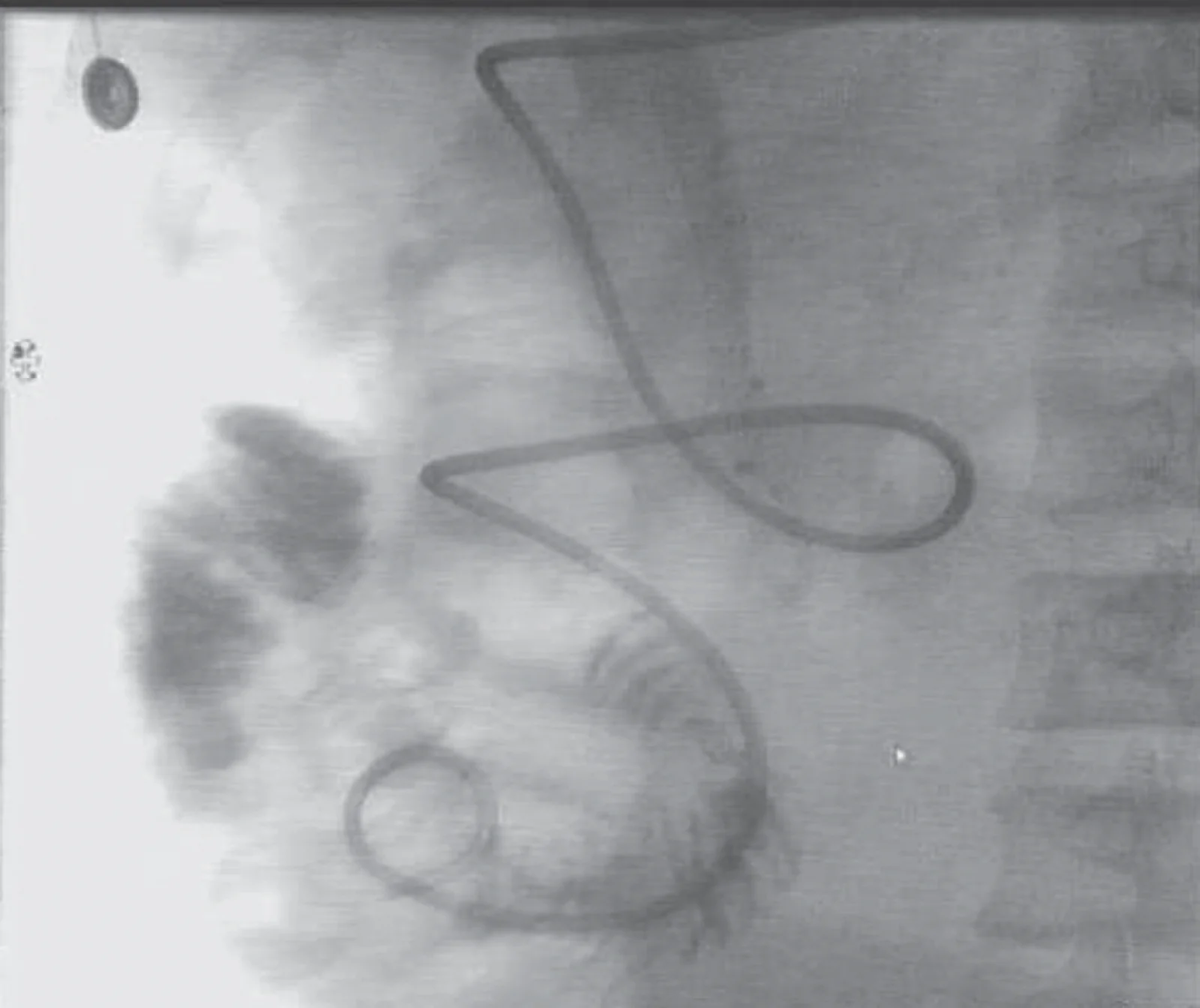
An X-ray image showing a covered metal biliary stent and an 8.5-Fr nasojejunal feeding tube.

The patient was managed conservatively with bowel rest, antibiotics, drainage, nutritional support, blood transfusions, and continuous renal replacement therapy. After approximately 3 months, oral intake was gradually resumed, and the patient was discharged in stable condition ( Video 1 ).

**Video 1** Conservative management was successfully performed for a delayed duodenal perforation following displacement and removal of a pancreatic duct stent. The video URI is as follows: .


This case highlights the need to recognize gastrointestinal perforation after the removal of a migrated pancreatic duct stent. Long-term dialysis may mask the typical manifestations of duodenal perforation. Duodenal perforation has traditionally been managed surgically, especially when peritoneal irritation or systemic inflammation is present. 1​ In this case, however, the perforation occurred more than 24 hours after the procedure and was successfully treated conservatively. To our knowledge, this is the first reported case after pancreatic stent removal and the first to be successfully managed without surgery.

Endoscopy_UCTN_Code_CPL_1AH_2AG

## Declaration of GenAI Use

ChatGPT (OpenAI) was used for language polishing.
